# Metabolic engineering of *Escherichia coli* for high-specificity production of isoprenol and prenol as next generation of biofuels

**DOI:** 10.1186/1754-6834-6-57

**Published:** 2013-04-24

**Authors:** Yanning Zheng, Qiang Liu, Lingling Li, Wen Qin, Jianming Yang, Haibo Zhang, Xinglin Jiang, Tao Cheng, Wei Liu, Xin Xu, Mo Xian

**Affiliations:** 1 CAS Key Laboratory of Biobased Materials, Qingdao Institute of Bioenergy and Bioprocess Technology, Chinese Academy of Sciences, No.189 Songling Road, Laoshan District, Qingdao, 266101, China; 2 University of Chinese Academy of Sciences, Beijing, 100049, China; 3 College of Food Science, Sichuan Agricultural University, Yaan, 625014, China

**Keywords:** Isoprenol, Prenol, Metabolic engineering, *Escherichia coli*, Biofuel

## Abstract

**Background:**

The isopentenols, including isoprenol and prenol, are excellent alternative fuels. However, they are not compounds largely accumulated in natural organism. The need for the next generation of biofuels with better physical and chemical properties impels us to develop biosynthetic routes for the production of isoprenol and prenol from renewable sugar. In this study, we use the heterogenous mevalonate-dependent (MVA) isoprenoid pathway for the synthesis of isopentenyl pyrophosphate (IPP) and dimethylallyl pyrophosphate (DMAPP) intermediates, and then convert IPP and DMAPP to isoprenol and prenol, respectively.

**Results:**

A mevalonate titer of 1.7 g/L was obtained by constructing an efficient MVA upper pathway in engineered *E. coli*. Different phosphatases and pyrophosphatases were investigated for their abilities in hydrolyzing the IPP and DMAPP. Consequently, ADP-ribose pyrophosphatase was found to be an efficient IPP and DMAPP hydrolase. Moreover, ADP-ribose pyrophosphatase from *Bacillus subtilis* (BsNudF) exhibited a equivalent substrate specificity towards IPP and DMAPP, while ADP-ribose pyrophosphatase from *E. coli* (EcNudF) presented a high substrate preference for DMAPP. Without the expression of any phosphatases or pyrophosphatases, a background level of isopentenols was synthesized. When the endogenous pyrophosphatase genes (*EcNudF* and *yggV*) that were capable of enhancing the hydrolyzation of the IPP and DMAPP were knocked out, the background level of isopentenols was still obtained. Maybe the synthesized IPP and DMAPP were hydrolyzed by some unknown hydrolases of *E. coli*. Finally, 1.3 g/L single isoprenol was obtained by blocking the conversion of IPP to DMAPP and employing the BsNudF, and 0.2 g/L ~80% prenol was produced by employing the EcNudF. A maximal yield of 12% was achieved in both isoprenol and prenol producing strains.

**Conclusions:**

To the best of our knowledge, this is the first successful report on high-specificity production of isoprenol and prenol by microbial fermentation. Over 1.3 g/L isoprenol achieved in shake-flask experiments represents a quite encouraging titer of higher alcohols. In addition, the substrate specificities of ADP-ribose pyrophosphatases were determined and successfully applied for the high-specificity synthesis of isoprenol and prenol. Altogether, this work presents a promising strategy for high-specificity production of two excellent biofuels, isoprenol and prenol.

## Background

With increasing concerns about environmental problems and energy security, research interest has been aroused in the field of microbial production of fuels and chemicals from renewable sources [1-4]. Short chain alcohols (C2-C5) represent a category of primary biofuels. Compared with the ethanol (C2), a main biofuel in use today [5], the C5 alcohols have higher energy density and lower water miscibility [1,6]. Moreover, the branched-chain unsaturated isopentenols (isoprenol and prenol) possess higher octane numbers and better low-temperature fluidity than their straight-chain saturated counterpart. In addition, by removing a molecule of water, isoprenol can be converted to isoprene, a very important monomer for synthesizing rubber. Global rubber demand is forecast to reach 27.2 million metric tons in 2012. Prenol is also of great importance as a building block to synthesize pyrethroid, a widely used pesticide. However, the isoprenol and prenol, which are conventionally manufactured from petroleum-derived isobutene and formaldehyde, are not compounds largely accumulated in natural organism. The need for the next generation of biofuels with better physical and chemical properties impels us to seek a biological route for direct production of isopentenols from renewable sugar in a microbial fermentation process.

**Figure 1:**
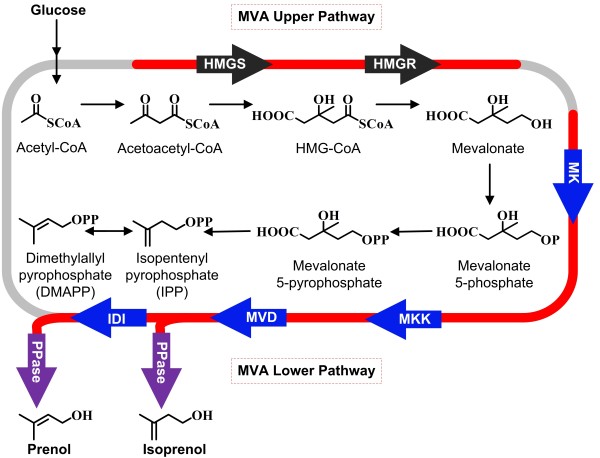
**Isoprenol and prenol biosynthesis pathway.** The heterogenous pathway that was introduced into *E. coli* is indicated by the red line. The black arrow represents the MVA upper pathway, the blue arrow represents the MVA lower pathway, and the purple arrow represents the isopentenol forming pathway. HMG-CoA, 3-hydroxy-3-methylglutaryl-coenzyme A; HMGS, HMG-CoA synthase; HMGR, HMG-CoA reductase; MK, mevalonate kinase; MKK, phosphomevalonate kinase; MVD, mevalonate pyrophosphate decarboxylase; IDI, isopentenyl pyrophosphate:dimethylallyl pyrophosphate isomerase.

To obtain the C5 backbones, we paid attention to the isoprenoid biosynthetic pathways, which exist as mevalonate-dependent (MVA) isoprenoid pathway in eukaryotes or deoxyxylulose 5-phosphate (DXP) pathway in plants and most prokaryotes [7]. The MVA and DXP pathways both end with the synthesis of isopentenyl pyrophosphate (IPP) and dimethylallyl pyrophosphate (DMAPP), which are used as the C5 building blocks for preparing isoprenoids *in vivo*[7-10]. Expression of the heterologous MVA pathway can avoid the unknown physiological control elements in *E. coli* and thus result in a high-level production of isoprenoid precursors [7]. Therefore, we took advantage of the MVA pathways for enhanced production of the precursors, IPP and DMAPP. The intermediates IPP and DMAPP can be finally converted to isoprenol and prenol by removing their pyrophosphates (Figure 1).

The recovery of the desired products will be greatly simplified if the single isoprenol or prenol can be harvested in the culture. However, thus far, no study has been carried out for high-specificity production of isoprenol and prenol in either wild-type or engineered microbes. To reach this goal, firstly, it is needed to employ phosphatases or pyrophosphatases with appropriate substrate specificities. Secondly, it is also needed to adjust the carbon flux into IPP and DMAPP, which can be interconverted with the help of isopentenyl pyrophosphate isomerase [11]. But unfortunately, no such efforts were made to tailor the composition of the isopentenols.

In this study, we devised a biosynthetic approach to selectively produce isoprenol and prenol in engineered *E. coli*. To improve the production of isoprenol and prenol, we compared the efficacies of different phosphatases and pyrophosphatases, and selectively directed the metabolic flux into the isoprenol versus prenol by inactivation of the heterogenous isopentenyl pyrophosphate isomerase or overexpression of a selective pyrophosphatase. We also discussed the effect of different pyrophosphatases on the hydrolyzation of IPP and DMAPP.

## Results

### Enhanced production of mevalonate

The MVA pathway can be divided into upper pathway and lower pathway, which are connected by mevalonate (Figure 1). To convert glucose to mevalonate, it is needed to introduce the exogenous HMG-CoA synthase and HMG-CoA reductase into the *E. coli* cell. We first expressed the HMG-CoA synthase (*HMGS*) and the truncated HMG-CoA reductase (t*HMGR*) from *Saccharomyces cerevisiae*[12], but the resultant engineered strain only produced 36.7 mg/L mevalonate. Tabata and Hashimoto improved the production of mevalonate by expressing a mevalonate pathway derived from *Enterococcus faecalis*[13]. To further enhance the synthesis of mevalonate in our study, we next employed the *mvaS* and *mvaE* from *E. faecalis* as the HMG-CoA synthase and HMG-CoA reductase, respectively. The yielded strain had a 45-fold increase in mevalonate accumulation, reaching a titer of 1.7 g/L. The improved mevalonate production may be attributed to the additional acetyl-CoA acetyltransferase activity of MvaE, which can promote the conversion of acetyl-CoA to acetoacetyl-CoA [14].

### High-specificity production of isoprenol and prenol

**Figure 2:**
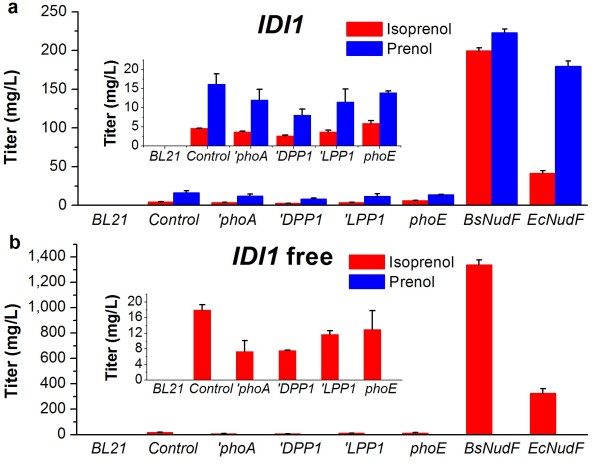
**Isoprenol and prenol production by employing different phosphatases and pyrophosphatases. a**, strains with the expression of *S. cerevisiae* isopentenyl pyrophosphate:dimethylallyl pyrophosphate isomerase gene *IDI1*; **b**, strains without the expression of *IDI1*. *BL21*, the wild-type *E. coli* BL21(DE3); *Control*, without the overexpression of any phosphatase or pyrophosphatase gene; ‘*phoA*, phosphatase gene ‘*phoA* from *E. coli*; ‘*DPP1* and *‘LPP1*, phosphatase genes ‘*DPP1* and *‘LPP1* from *S. cerevisiae*; *phoE*, phosphatase gene from *B. subtilis*; *BsNudF*, the ADP-ribose pyrophosphatase gene from *B. subtilis*; *EcNudF*, the ADP-ribose pyrophosphatase gene from *E. coli*. The error bars represent the range from three independent experiments.

The conversion of mevalonate to IPP and DMAPP requires four steps, sequentially catalyzed by mevalonate kinase, phosphomevalonate kinase, mevalonate pyrophosphate decarboxylase and isopentenyl pyrophosphate: dimethylallyl pyrophosphate isomerase, whose coding genes in *S. cerevisiae* S288c are *ERG12*, *ERG8*, *ERG19* and *IDI1*, respectively [15-18]. We cloned the four genes of *S. cerevisiae* into the pTrcHis2B to generate pTrcLower expression vector, with a method of successive hybridization [19]. Expression of the whole MVA pathway in *E. coli* (control strain) resulted in 4.6 mg/L isoprenol and 16.2 mg/L prenol (Figure 2a, *Control*).

The low level of isopentenol production is probably attributed to the poor phosphatase or pyrophosphatase activity in *E. coli*. Therefore, it is needed to overexpress phosphatase or pyrophosphatase that can efficiently hydrolyze the phosphoester bond of IPP or DMAPP. Phosphatases DPP1 and LPP1 from *S. cerevisiae* were found to be able to promote the production of geranylgeraniol by removing the pyrophosphatases of geranylgeranyl diphosphate [20]. The alkaline phosphatase is a nonspecific phosphomonoesterase that can remove the inorganic phosphate to release corresponding alcohols [21]. It is possible that these phosphatases also have catalytic activities towards IPP and DMAPP. So we tested the efficiencies of the phosphatases ‘PhoA (a leaderless version of PhoA) from *E. coli*, ‘DPP1 (a leaderless version of DPP1) from *S. cerevisiae*, ‘LPP1 (a leaderless version of LPP1) from *S. cerevisiae* and PhoE from *B. subtilis* on the production of isopentenols. The engineered strains employing the four phosphatases still only produced the background level of isopentenols (Figure 2, *‘phoA*, *‘DPP1*, *‘LPP1* and *phoE*), suggesting that these phosphatases can not efficiently remove the pyrophosphates of IPP and DMAPP.

So we further introduced the ADP-ribose pyrophosphatase gene from *B. subtilis* (*BsNudF*) into the strain that employed the whole MVA pathway [22]. The resultant *BsNudF* expressing strain dramatically enhanced the synthesis of isoprenol and prenol (Figure 2a, *BsNudF*). This result demonstrates that the ADP-ribose pyrophosphatase is a good candidate biocatalyst to remove the pyrophosphate from IPP and DMAPP.

**Figure 3:**
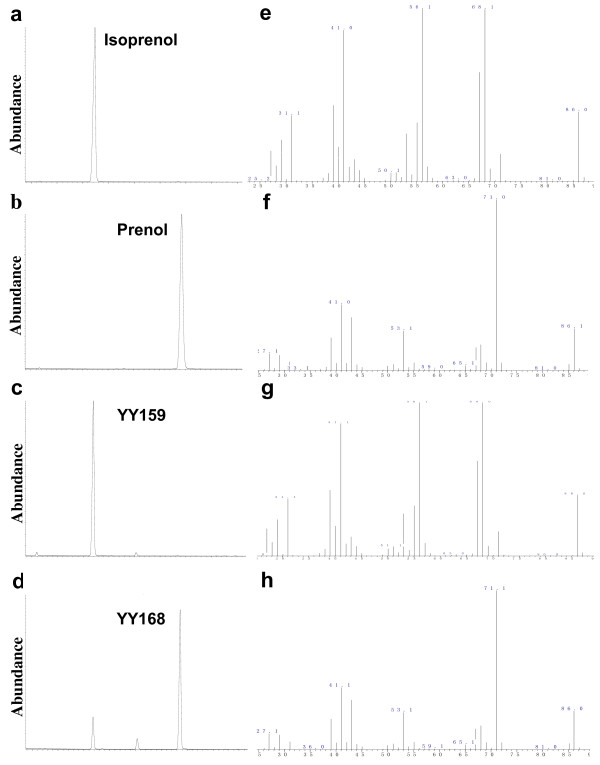
**GC-MS analysis of isoprenol and prenol in the cultures.** By comparing with the authoritative isoprenol (**a, e**) and prenol (**b, f**), the capacities of isoprenol and prenol biosynthesis were verified in the engineered strains YY159 and YY168, respectively. YY159 produced the isoprenol as its single product (**c**, **g**), while YY168 produced a blend of isoprenol and prenol, with prenol as the dominant component (**d**, **h**). **a**, **b**, **c**, **d**, total ion chromatogram (TIC); **e**, **f**, **g**, **h**, mass spectrum.

We noticed that expression of *BsNudF* with the whole MVA pathway (YY158) yielded a blend of isoprenol and prenol in the ratio of ~1:1 (Figure 2a, *BsNudF*), suggesting that BsNudF has high affinity for both IPP and DMAPP. The recovery of the desired products will be greatly simplified if the single isoprenol or prenol can be harvested in the culture. It is needed for us to tailor the composition of the products by further metabolic engineering. To obtain a single isoprenol, we blocked the conversion of IPP to DMAPP by deleting the *IDI1* gene of the pTrcLower. Indeed, the strains without the expression of *IDI1* synthesized the isoprenol as their single product (Figure 2b). Finally, an engineered strain expressing *BsNudF* and partial MVA pathway (YY159) produced 1,337 mg/L isoprenol in shake flask, with prenol nearly undetected (Figure 2b, *BsNudF*; Figure 3). This result demonstrates that the metabolic flux of IPP to DMAPP was successfully blocked, and nearly all synthesized IPP entered the isoprenol biosynthetic pathway, contributing to the greatly improved isoprenol production.

To raise the specificity for prenol production, it is needed to find a pyrophosphatase that has a higher substrate preference towards DMAPP. As above mentioned, the strain without expression of any phosphatase and pyrophosphatase produced 20.8 mg/L isoprenol and prenol, with the main contribution coming from prenol (Figure 2a, *Control*). This result suggests some endogenous pyrophosphatase of *E. coli* probably contributed to the background level of isopentenols, given the expression of phosphatase didn’t exhibit any enhanced production of isopentenols. In addition, expression of ADP-ribose pyrophosphatase from *B. subtilis* (*BsNudF*) greatly enhanced the production of isopentenols as aforementioned. Therefore, we paid attention to the ADP-ribose pyrophosphatase of *E. coli* (encoded by *EcNudF* gene). By overexpressing the *EcNudF* and the entire MVA pathway (YY168), 0.2 g/L isopentenols were finally achieved, with prenol accounting for over 80% (Figure 2a, *EcNudF*; Figure 3).

### Knockout of the endogenous pyrophosphatase genes of *E. coli*

As above mentioned, isopentenols can be synthesized in small amounts even if no pyrophosphatase was overexpressed. We speculated the ADP-ribose pyrophosphatases of *E. coli* (EcNudF) contributed to the background level of isopentenols, given the overexpression of *EcNudF* notably enhanced the production of isopentenols. However, when the endogenous *EcNudF* gene of *E. coli* was knocked out, the background level of isopentenols was still observed.

**Figure 4:**
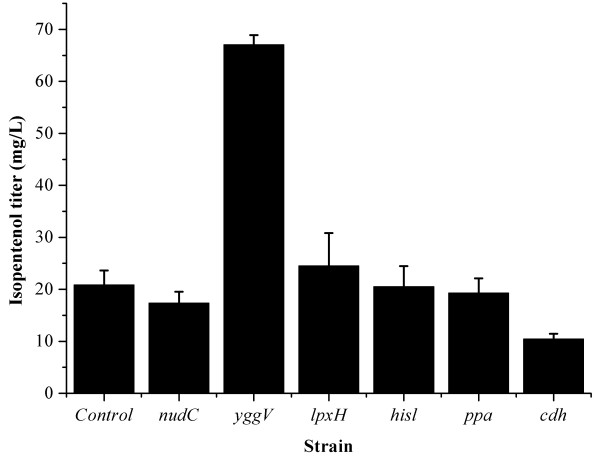
**Isopentenol production by employing different pyrophosphatases of *E. coli *except ADP-ribose pyrophosphatase.***Control*, ISP2148 strain; *nudC*, strain expressing NADH pyrophosphatase; *yggV*, strain expressing dITP/XTP pyrophosphatase; *lpxH*, strain expressing UDP-2,3-diacylglucosamine pyrophosphatase; *hisl*, strain expressing phosphoribosyl-ATP pyrophosphatase; *ppa*, strain expressing inorganic pyrophosphatase; *cdh*, strain expressing CDP-diacylglycerol pyrophosphatase.

To test if other pyrophosphatases are also capable of hydrolyzing the IPP and DMAPP, we overexpressed *E. coli* NADH pyrophosphatase (*nudC*), dITP/XTP pyrophosphatase (*yggV*), UDP-2,3-diacylglucosamine pyrophosphatase (*lpxH*), phosphoribosyl-ATP pyrophosphatase (*hisl*), inorganic pyrophosphatase (*ppa*) and CDP-diacylglycerol pyrophosphatase (*cdh*) in ISP2148 strain, respectively. Overexpression of *yggV* improved the isopentenols to some extent, while overexpression of other pyrophosphatases did not enhance the production of isopentenols (Figure 4). It demonstrates that dITP/XTP pyrophosphatase is also able to hydrolyze the pyrophosphates of IPP and DMAPP.

We further deleted the *yggV* gene in the *EcNudF* gene knockout mutant, yielding a *EcNudF-yggV* double-gene knockout mutant. However, expression of the MVA pathway in this double-gene knockout mutant still synthesized a background level of isopentenols. To check if the background level of isopentenols was produced by the spontaneous hydrolysis of IPP and DMAPP, we determined the IPP and DMAPP hydrolysis in an enzyme-free solution. However, no isopentenols was formed after incubating the solution for the same time as the shake-flask experiment. Therefore, it is speculated that some unknown *E. coli* hydrolases resulted in the background level of isopentenols.

### Effect of mevalonate accumulation on the production of isopentenols

It has been known that mevalonate is an important intermediate in the isopentenol biosynthetic pathways. The isopentenol production may be further improved by accelerating the synthesis of mevalonate. It was found that the native HMG-CoA synthase MvaS had a non-optimal catalytic efficiency. When the Ala-110 of the MvaS was replaced by glycine, the mutated enzyme MvaSA110G achieved a 140-fold increase in overall reaction rate [23]. In addition, the biosynthesis of isoprenoid compounds was found to be proportional to the amount of mevalonate, up to a concentration of over 5 g/L mevalonate [7].

**Figure 5:**
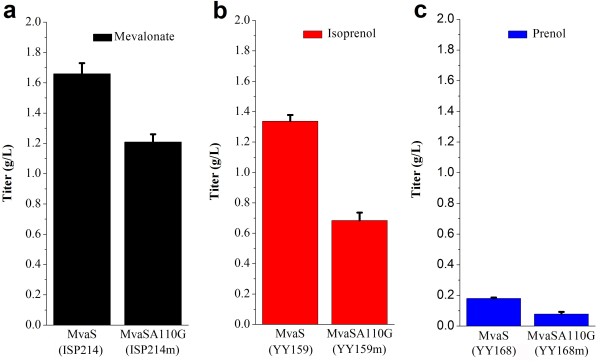
**The effect of a mutated HMG-CoA synthase (MvaSA110G) on the production of mevalonate, isoprenol and prenol.** The strain employing MvaSA110G (ISP214m) produced a lower titer of mevalonate than the strain carrying the native enzyme MvaS (ISP214) (**a**). And in the meanwhile, the decreased production of isoprenol (**b**) or prenol (**c**) was respectively obtained in two strains expressing the MvaSA110G (YY159 and YY168). The error bars represent the range from two independent experiments.

Therefore, we substituted the MvaS with the MvaSA110G as the HMG-CoA synthase to further improve the production of mevalonate. But unexpectedly, the strain harboring the MvaSA110G (ISP214m) only produced 1.23 g/L mevalonate, decreased by 25.5% when compared with the strain carrying MvaS (ISP214) (Figure 5a). The reduced supply of mevalonate directly led to the decreased output of isoprenol and prenol. The isoprenol and prenol production decreased to 0.7 g/L (YY159m) and 0.08 g/L (YY168m), respectively (Figure 5b; 5c). It is surprising that the accelerated reaction rate of MvaS did not enhance the production of both mevalonate and isopentenols. A future metabolic flux analysis will make this conversion process clearer.

### Evaluation of the isopentenol production of two optimized strains

**Figure 6:**
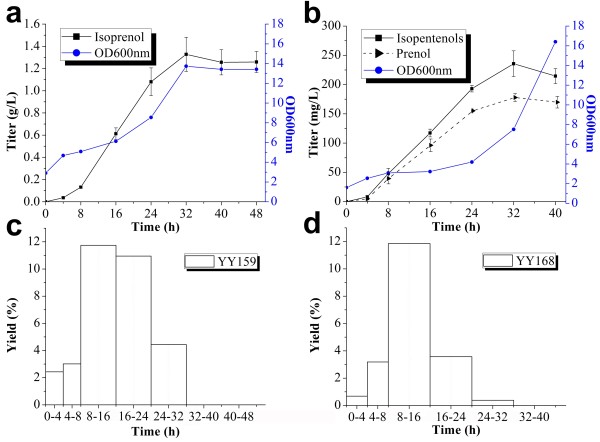
**Characterization of isopentenol production of two best-performed strains YY159 and YY168. YY159, expressing pyrophosphatase BsNudF and the whole MVA pathway except IDI1; YY168, expressing pyrophosphatase EcNudF and the whole MVA pathway.** YY159 produced a single isoprenol, while YY168 produced a major prenol. **a**, the isoprenol titer and growth curve of YY159; **b**, the isopentenol titers and growth curve of YY168; **c**, the isoprenol yield of YY159; **d**, the isopentenol yield of YY168.

Thus far, YY159 and YY168 are the best-performed strains for the production of isoprenol and prenol, respectively. To better understand their isopentenol production, we determined their product titers and yields in specified periods of time. YY159 and YY168 shared a similar process in the production of isopentenols. They both reached their highest isopentenol titers approximately 32 h post-induction (Figure 6a; 6b). The linear isoprenol productivity of YY159 and prenol productivity of YY168 appeared approximately 4 ~ 8 h post-induction, constant at 59.3 mg/L/h and 9.2 mg/L/h, respectively. In addition, the maximal isopentenol yield of 12% (i.e. 0.12 g isopentenols/g glucose consumed) was observed in both YY159 and YY168 (Figure 6c; 6d).

## Discussion

In this study, we present a microbial system for high-specificity production of isoprenol and prenol, two promising new generation of biofuels, from sugar feedstocks. It mainly employs the MVA pathway that is used for isoprenoid biosynthesis in nature [7,8], and finally directs the IPP or DMAPP intermediate to pyrophosphate hydrolization pathway for the production of isoprenol or prenol. This endeavor met with several major challenges: the construction of an efficient MVA pathway to IPP and DMAPP from central metabolic intermediates, the exploration of phosphatases or pyrophosphatases with appropriate substrate specificities, and the engineering of the host *E. coli* metabolism to selectively channel carbon flux and energy into isoprenol or prenol biosynthetic pathway. The challenges met here are likely to be characteristic for high-specificity production of any isomers or homologues biologically, as these compounds are quite similar in structure. Our provided approaches to these problems may contribute to biocatalytic production of more challenging relevant targets.

Our success in respectively producing over 1.3 g/L single isoprenol and 0.2 g/L ~80% prenol from two engineered *E. coli* strains demonstrates this strategy is promising for the production of next generation of biofuels. Though it is still needed to further improve the production of the isoprenol and prenol by detailed optimization of the isopentenol biosynthetic pathways, our results are encouraging from several standpoints.

First, we achieved the production of isoprenol and prenol with high specificity. To our knowledge, this is the first successful report on high-specificity production of isoprenol and prenol from renewable sugar.

Second, we constructed and expressed an efficient isopentenol biosynthetic pathway. Over 1.3 g/L isoprenol achieved in lab-scale shake-flask experiments is a quite encouraging isopentenol titer, demonstrating that this strategy is promising for the commercial production of higher-alcohol biofuels.

Third, we found that the ADP-ribose pyrophosphatase from different organism had varied substrate preferences towards IPP and DMAPP. In earlier study, no attention was paid to the substrate specificity of ADP-ribose pyrophosphatase. Our finding opens the door to tailoring the composition of isopentenols by employing different pyrophosphatase. Developing pyrophosphatase with higher hydrolyzing efficiency and specificity may contribute to a further improvement of the isopentenol production.

Fourth, the yield of 12% achieved in shake flask was observed during the post-induction, linear isopentenol production period. This is a rather high yield, even slightly higher than the well developed 3-methyl-1-butanol production [6]. A further yield improvement will be obtained under controlled conditions by using a scalable fermentation system.

Our data also suggests that mevalonate is an important metabolite in determining the production of isoprenol, given that over 80% mevalonate can be converted to isoprenol. It provides another clue to further increase the production of isoprenol.

**Figure 7:**
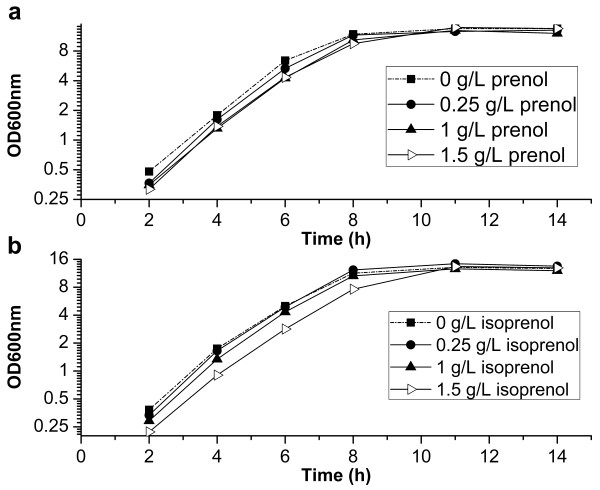
**The inhibitory effect of isoprenol and prenol on the growth of *E. coli*.** The *E. coli* BL21(DE3) was cultured at 37°C in media supplemented with increasing concentrations of exogenous prenol or isoprenol. OD600nm, optical density at 600 nm.

Compared with the isoprenol production, a much lower titer of prenol was obtained. The lower biomass probably resulted in the decreased prenol production, given the prenol producing strain (YY168) did not grow so well as the strain for isoprenol synthesis (YY159). Though YY168 finally obtained a higher biomass than YY159, YY168 had lower biomasses during the period when the isopentenols were largely produced (Figure 6). To check if the products prenol and isoprenol inhibit the normal growth of *E. coli*, we measured the growth of *E. coli* BL21(DE3) in media supplemented with increasing concentrations of exogenous prenol or isoprenol. A slight growth inhibition was indeed observed in the presence of prenol, but the inhibitory effect of prenol on the growth of *E. coli* was even weaker than that of isoprenol (Figure 7). Moreover, the strain YY168 produced a much lower product titer than YY159, which grew well during the production of isoprenol. Therefore, the growth inhibition of strain YY168 was not caused by the synthesized prenol.

Martin *et al.* found that, using mevalonate as substrate, the expression of partial MVA lower pathway (MK, MKK and MVD) resulted in the highest concentration of IPP, the expression of entire MVA lower pathway (MK, MKK, MVD and IDI) led to a decreased concentration of IPP, and the expression of an additional synthetic pathway that converts IPP and DMAPP to other metabolites further decreased the intracellular concentration of IPP [7]. That is to say, IPP will largely accumulate in engineered *E. coli* if it can’t be efficiently converted to other metabolites. In the meanwhile, compared to BsNudF, EcNudF has a much poorer substrate specificity towards IPP, making IPP can’t be efficiently hydrolyzed. Therefore, the lower cell density of YY168 may be attributed to the accumulation of IPP, which is toxic and can inhibit normal cell growth [7].

## Conclusions

The engineering strategy described above inaugurates a new realm for the production of alcoholic biofuels. The substrate specificities of ADP-ribose pyrophosphatases were determined and successfully applied for the high-specificity synthesis of isoprenol and prenol. The desired isoprenol and prenol were finally produced with high specificities, being the first successful report on high-specificity production of isoprenol and prenol from renewable sugar. The achieved over 1.3 g/L isoprenol represents an encouraging isopentenol titer. In addition, the isopentenols can be isolated with the well-developed butanol recovery techniques, such as gas stripping. Therefore, this strategy provides the potential and possibility for scale-up production of isopentenols by utilizing the butanol production line without too much reconstruction. Altogether, this work presents a promising strategy for high-specificity production of two excellent biofuels, isoprenol and prenol.

## Methods

### Plasmid construction

The *HMGS* [Genbank: NM_001182489] was polymerase chain reaction (PCR)-amplified from genomic DNA of *S. cerevisiae* (ATCC 204508) with the primer set HMGS-F and HMGS-R. The PCR product digested with NdeI and XhoI was cloned into pACYCDuet-1 (Novagen, Darmstadt, Germany) cut with the same restriction enzymes, creating pISP211. The t*HMGR* (a truncated version of *HMGR*) was PCR-amplified from genomic DNA of *S. cerevisiae* (ATCC 204508) with the primer set tHMGR-F and tHMGR-R [7,12]. The PCR product digested with NcoI and BamHI was cloned into pISP211 cut with the same restriction enzymes, creating pISP212.

The *mvaE* [Genbank: AF290092] was PCR-amplified from genomic DNA of *Enterococcus faecalis* (ATCC 700802D-5) with the primer set mvaE-F and mvaE-R. The PCR product digested with NcoI and BamHI was cloned into pACYCDuet-1 cut with the same restriction enzymes, creating pISP213. The *mvaS* [Genbank: AF290092] was PCR-amplified from genomic DNA of *E. faecalis* (ATCC 700802D-5) with the primer set mvaS-F and mvaS-R. The PCR product digested with SacI and PstI was cloned into pISP213 cut with the same restriction enzymes, creating pISP214.

The *ERG12*, *ERG8*, *ERG19* and *IDI1* genes from *S. cerevisiae* (ATCC 204508) were cloned into pTrcHis2B (Invitrogen, Carlsbad, CA) using a method of successive hybridization to yield pTrcLower [19].

To delete the *IDI1* gene from pTrcLower, a fragment containing *ERG19*, *ERG8* and a part of *ERG12* was first PCR-amplified from pTrcLower with the primers IDIKO-F and IDIKO-R, then digested with BamHI and PstI, and finally cloned into the pTrcLower cut with the same restriction enzymes. The resultant recombinant plasmid designated pISP9.

The ‘*phoA* (a leaderless version of *phoA* [Genbank: NC_000913.2:400971..402386]) was PCR-amplified from genomic DNA of *E. coli* K12 with the primer set phoA-F2 and phoA-R. The PCR product digested with BglII and XhoI was cloned into pCOLADuet-1 (Novagen, Darmstadt, Germany) cut with the same restriction enzymes, creating pYY11.

The *‘DPP1* (a leaderless version of *DPP1* [Genbank: NM_001180592]) was PCR-amplified from genomic DNA of *S. cerevisiae* (ATCC 204508) with the primer set DPP1-F2 and DPP1-R. The PCR product digested with BglII and XhoI was cloned into pCOLADuet-1 cut with the same restriction enzymes, creating pYY12.

The *‘LPP1* (a leaderless version of *LPP1* [Genbank: NM_001180811]) was PCR-amplified from genomic DNA of *S. cerevisiae* (ATCC 204508) with the primer set LPP1-F2 and LPP1-R. The PCR product digested with BglII and XhoI was cloned into pCOLADuet-1 cut with the same restriction enzymes, creating pYY13.

The *phoE* [Genbank: AL009126] was PCR-amplified from genomic DNA of *Bacillus subtilis* (ATCC 23857) with the primer set phoE-F and phoE-R. The PCR product digested with BglII and XhoI was cloned into pCOLADuet-1 cut with the same restriction enzymes, creating pYY14.

The *BsNudF* [Genbank: AL009126] was PCR-amplified from genomic DNA of *B. subtilis* (ATCC 23857) with the primer set BsNudF-F and BsNudF-R. The PCR product digested with NcoI and BamHI was cloned into pCOLADuet-1 cut with the same restriction enzymes, creating pYY15.

The *EcNudF* [Genbank: NC_010473.1:3273048..3273677] was PCR-amplified from genomic DNA of *E. coli* K12 with the primer set EcNudF-F and EcNudF-R. The PCR product digested with NcoI and BamHI was cloned into pCOLADuet-1 cut with the same restriction enzymes, creating pYY16.

The *nudC*, *yggV*, *hisl*, *lpxH*, *ppa* and *cdh* were PCR-amplified from genomic DNA of *E. coli* K12 with the primer set nudC-F/nudC-R, yggV-F/yggV-R, hisl-F/hisl-R, lpxH-F/lpxH-R, ppa-F/ppa-R and cdh-F/cdh-R, respectively. The PCR products digested with NcoI and BamHI were cloned into pCOLADuet-1 cut with the same restriction enzymes, creating pYY26, pYY36, pYY46, pYY56, pYY66 and pYY76, respectively.

All the plasmids and strains used in this work are listed in Table 1, and the oligonucleotide primers are given in Table 2.

**Table 1:** Bacterial strains and plasmids used in this study

**Plasmid or strain**	**Relevant genotype or description**	**Reference**
Plasmids		
pACYCDuet-1	P15A origin; Cm^R^; P_T7_	Novagen
pTrcHis2B	ColE1 origin; Amp^R^; P_trc_	Invitrogen
pCOLADuet-1	ColA origin; Kan^R^; P_T7_	Novagen
pISP212	P15A origin; Cm^R^; P_T7_::*HMGS*(Sc)-t*HMGR*(Sc)	This study
pISP214	P15A origin; Cm^R^; P_T7_::*mvaS*(Ef)-*mvaE*(Ef)	This study
pISP214m	P15A origin; Cm^R^; P_T7_::*mvaS*A110G-*mvaE*(Ef)	This study
pTrcLower	ColE1 origin; Amp^R^; P_trc_:: *ERG12*(Sc)-*ERG8*(Sc)-*ERG19*(Sc)-*IDI1*(Sc)	[19]
pISP9	ColE1 origin; Amp^R^; P_trc_:: *ERG12*(Sc)-*ERG8*(Sc)-*ERG19*(Sc)	This study
pYY11	ColA origin; Kan^R^; P_T7_:: ‘*phoA*	This study
pYY12	ColA origin; Kan^R^; P_T7_:: ‘*DPP1*	This study
pYY13	ColA origin; Kan^R^; P_T7_:: ‘*LPP1*	This study
pYY14	ColA origin; Kan^R^; P_T7_:: *phoE*	This study
pYY15	ColA origin; Kan^R^; P_T7_::*BsNudF*	This study
pYY16	ColA origin; Kan^R^; P_T7_::*EcNudF*	This study
pYY26	ColA origin; Kan^R^; P_T7_::*NudC*	This study
pYY36	ColA origin; Kan^R^; P_T7_::*yggV*	This study
pYY46	ColA origin; Kan^R^; P_T7_::*hisl*	This study
pYY56	ColA origin; Kan^R^; P_T7_::*lpxH*	This study
pYY66	ColA origin; Kan^R^; P_T7_::*ppa*	This study
pYY76	ColA origin; Kan^R^; P_T7_::*cdh*	This study
Strains		
DH5α	*deoR*, recA1, endA1, hsdR17(rk^-^,mk^+^), phoA, supE44, λ-, thi^-^1, gyrA96, relA1	Takara
BL21(DE3)	*E. coli B dcm ompT hsdS*(r_B_^-^m_B_^-^) *gal*	Invitrogen
W 3110	F^-^ λ^-^ rph-1 INV(rrnD, rrnE)	[24]
DNF(DE3)	W3110: ∆*EcNudF*(DE3)	This study
DNFYV(DE3)	W3110: ∆*EcNudF*∆*yggV*(DE3)	This study
ISP212	BL21(DE3) harboring pISP212	This study
ISP214	BL21(DE3) harboring pISP214	This study
ISP214m	BL21(DE3) harboring pISP214m	This study
ISP2148	BL21(DE3) harboring pISP214 and pTrcLower	This study
ISP2149	BL21(DE3) harboring pISP214 and pISP9	This study
YY118	BL21(DE3) harboring pISP214, pTrcLower and pYY11	This study
YY128	BL21(DE3) harboring pISP214, pTrcLower and pYY12	This study
YY138	BL21(DE3) harboring pISP214, pTrcLower and pYY13	This study
YY148	BL21(DE3) harboring pISP214, pTrcLower and pYY14	This study
YY158	BL21(DE3) harboring pISP214, pTrcLower and pYY15	This study
YY168	BL21(DE3) harboring pISP214, pTrcLower and pYY16	This study
YY119	BL21(DE3) harboring pISP214, pISP9 and pYY11	This study
YY129	BL21(DE3) harboring pISP214, pISP9 and pYY12	This study
YY139	BL21(DE3) harboring pISP214, pISP9 and pYY13	This study
YY149	BL21(DE3) harboring pISP214, pISP9 and pYY14	This study
YY159	BL21(DE3) harboring pISP214, pISP9 and pYY15	This study
YY169	BL21(DE3) harboring pISP214, pISP9 and pYY16	This study
YY159m	BL21(DE3) harboring pISP214m, pISP9 and pYY15	This study
YY168m	BL21(DE3) harboring pISP214m, pTrcLower and pYY16	This study
YY268	BL21(DE3) harboring pISP214, pTrcLower and pYY26	This study
YY368	BL21(DE3) harboring pISP214, pTrcLower and pYY36	This study
YY468	BL21(DE3) harboring pISP214, pTrcLower and pYY46	This study
YY568	BL21(DE3) harboring pISP214, pTrcLower and pYY56	This study
YY668	BL21(DE3) harboring pISP214, pTrcLower and pYY66	This study
YY768	BL21(DE3) harboring pISP214, pTrcLower and pYY76	This study

**Table 2:** Primers used in this study

**Name**	**Sequence (5′ → 3′)**
HMGS-F	GGAATTCCATATGAAACTCTCAACTAAACTTTG
HMGS-R	CACCTCGAGTTATTTTTTAACATCGTAAGATC
tHMGR-F	CATGCCATGGACCAATTGGTGAAAACTGAAG
tHMGR-R	CGGGATCCTTAGGATTTAATGCAGGTGACG
mvaS-F	CCAGAGCTCAGGAGGTAAAAAAACATGACAATTGGGATTGATAAAATTA
mvaS-R	CAACTGCAGTTAGTTTCGATAAGAGCGAACG
mvaE-F	CATGCCATGGAGGAGGTAAAAAAACATGAAAACAGTAGTTATTATTGATGC
mvaE-R	CGCGGATCCTTATTGTTTTCTTAAATCATTTAAAATAG
IDIKO-F	GTCTTTTGGATCCGTTGTTAGCTC
IDIKO-R	CAAAACTGCAGTTATTCCTTTGGTAGACCAGTC
phoA-F2	CTCAGATCTCCGGACACCAGAAATGCCTGTTC
phoA-R	CACCTCGAGTTATTTCAGCCCCAGAGCGGC
DPP1-F2	CTCAGATCTCCAACCGTTCGAACGTCAGTTTTAC
DPP1-R	CACCTCGAGTTACATACCTTCATCGGACAAAG
LPP1-F2	CTCAGATCTCGAGATATCCCTGGTACCTAGGG
LPP1-R	CACCTCGAGTTACTAAACACTAACCGGTGAAGGAAG
phoE-F	CTAGCCATGGGCATGACAGCCGTTTGTTTAGTAAG
phoE-R	CAGGGATCCTTATTTGATAAAGCCGGATAAGTG
BsNudF-F	CTAGCCATGGGCATGAAATCATTAGAAGAAAAAAC
BsNudF-F	CAGGGATCCTCATTTTTGTGCTTGGAGCG
EcNudF-F	CTAGCCATGGGCATGCTTAAGCCAGACAACCTG
EcNudF-F	CAGGGATCCTTATGCCCACTCATTTTTTAACG
nudC-F	CTAGCCATGGATCGTATAATTGAAAAATTAG
nudC-R	CAGGGATCCTCACTCATACTCTGCCCGACAC
yggV-F	CTAGCCATGGGCATGCAAAAAGTTGTCCTCGCAAC
yggV-R	CAGGGATCCTTAACCATTACGTAAAGCGTCC
hisl-F	CTAGCCATGGGCATGTTAACAGAACAACAACGTCG
hisl-R	CAGGGATCCTCACTGATGCCGTTTACGCAG
lpxH-F	CTAGCCATGGCGACACTCTTTATTGCAG
lpxH-R	CAGGGATCCTTAAAACGGAAAATGAATCAGCTC
ppa-F	CTAGCCATGGGCATGAGCTTACTCAACGTCCCTG
ppa-R	CAGGGATCCTTATTTATTCTTTGCGCGCTCG
cdh-F	CTAGCCATGGGCATGAAAAAAGCGGGTCTTCT
cdh-R	CAGGGATCCTTAACGCAAAATCTCACACTG
KnudF-F	TCGCTGAAATTCACATTTAATTCACTATTAGTGCCAGGACATTAACAATGCATGGGAATTAGCCATGGTCC
KnudF-R	TCAGGAAAGTCAGGTGTGTAACGCTTCATTTATGCCCACTCATTTTTTAATGTAGGCTGGAGCTGCTTCG
KyggV-F	GCAACAAATCCCGCCAGAAATCGCGGCGTTAATTAATTAGGTATCCTATGCATGGGAATTAGCCATGGTCC
KyggV-R	TGTGAATGTAGAGACTCAGCGGAGGTAATTTAACCATTACGTAAAGCGTCTGTAGGCTGGAGCTGCTTCG
KnudF-vF	ACCCAGAAAGGCTCAGGCCG
KnudF-vR	ACAGTTTCGCCGGGTGCGTC
KyggV-vF	GACGGCCAGGCCAACAGTCA
KyggV-vR	CGGCCCTGAGCGTAAGCCAC
A110G-F	CTCTTTCGAAATCAAGGAAGGTTGTTACGGAGC
A110G-R	CCTTCCTTGATTTCGAAAGAGCGAGCGAAAG

Underlines indicate restriction enzyme sites.

### Site-directed mutagenesis

A method based on the amplification of the entire plasmid using primers that include the desired changes was employed for the site-directed mutagenesis [25]. The Ala-110 of HMG-CoA synthase MvaS was mutated to Gly by replacing a nucleotide C with G using the mutant primers A110G-F and A110G-R.

The PrimeSTAR HS DNA polymerase (Takara) was used for PCR. The PCR conditions were as follows: 98°C for 30 sec; 12 × (98°C for 5 sec; 63°C for 5 sec; 72°C for 8 min); 72°C for 10 min. The mutant was verified by sequencing (BGI).

### Gene knockout and λDE3 lysogenization

The *EcNudF* gene of *E. coli* was knocked out using the one-step inactivation method previously reported [26]. This method includes following steps: amplification of the kanamycin-resistant (Kan^R^) gene with PCR using pKD4 as template. The PCR product was transformed into the cells by electro-poration after gel purification. Transformants were selected with kanamycin-resistance plate. The mutant was verified by PCR using the test primers KOnudF-VF/KOnudF-VR. The kanamycin cassette was removed with the helper plasmid pCP20 that expresses FLP. The *E. coli* DNF was finally obtained after removing the helper plasmids.

To express target genes cloned in vectors under the control of the T7 promoter, λDE3 prophage was integrated into the *E. coli* DNF chromosome using the λDE3 lysogenization kit (Novagen, Darmstadt, Germany) according to the manufacturer’s instructions. The obtained strain was designated as *E. coli* DNF (DE3). The *yggV* gene was then deleted with the same method using *E. coli* DNF (DE3) as the starting strain. The resultant double-gene knockout mutant was designated as *E. coli* DNFYV (DE3).

### Bacterial strains, media and growth conditions

The bacterial strains used in this study are listed in Table 1. *E. coli* BL21(DE3) (Invitrogen, Carlsbad, CA) was used as the host to overproduce proteins. During strain construction, cultures were grown aerobically at 37°C in Luria Broth (10 g/L tryptone, 10 g/L NaCl, and 5 g/L yeast extract). Kanamycin (50 mg/L), Ampicillin (100 mg/L) or chloramphenicol (34 mg/L) was added if necessary. For initial production experiments in shake flasks, strains were grown in a medium consisted of the following: 7.5 g/L K_2_HPO_4_ · 3H_2_O, 2.1 g/L citric acid monohydrate, 0.3 g/L ferric ammonium citrate, 2.92 g/L (NH4)_2_SO_4_, 30 g/L of glucose, 9 g/L beef extract, 4 mM MgSO_4_, trace metals mix (2.86 mg/L H_3_BO_3_, 1.81 mg/L MnCl_2_ · 4H_2_O, 0.222 mg/L ZnSO_4_ · 7H_2_O, 0.39 mg/L Na_2_MoO_4_ · 2H_2_O, 0.079 mg/L CuSO_4_ · 5H_2_O, 49.4 μg/L Co(NO_3_)_2_ · 6H_2_O). The residual glucose was measured by the SBA-40D Biosensor equipped with glucose oxidase membrane electrodes (Shandong Academy of Sciences, Jinan, China). Protein production was induced with 0.5 mM isopropyl β-D-thiogalactoside (IPTG) at 30°C.

### Determination of mevalonate

Ten milliliter cell free cultures were collected, adjusted to pH 2.0 using hydrochloric acid, and incubated at 45°C for 1 hour. Then 5 g of anhydrous granular sodium sulphate was added to each vial followed by 10 ml of ethyl acetate. The vials were mixed on a vortexer for 5 min and the phases were allowed to separate naturally. One milliliter supernatant was collected for gas chromatograph (GC) analysis. The separation of mevolonatone lactone was performed using a CP-FFAP CB capillary column (25 m × 0.25 mm; 0.2 μm film thickness) purchased from Agilent Technologies (Santa Clara, CA). The oven temperature was initially held at 150°C for 1 min, then raised with a gradient of 10°C/min until reaching 250°C, and finally held at 250°C for 10 min. Nitrogen was used as the carrier gas. The injector and detector were held at 250°C and 270°C, respectively.

### Analysis of isoprenol and prenol by GC-MS

Isoprenol and prenol produced by the engineered strains were identified by GC–MS. The system consisted of model 7890A network GC system (Agilent Technologies) and a model 5975C network mass selective detector (Agilent Technologies, Santa Clara, CA). A HP-INNOWAX capillary column (30 m × 0.25 mm; 0.25 μm film thickness; Agilent Technologies) was used, with helium as the carrier gas. The following oven temperature program was carried out: 100°C for 1 min, increase of 5°C/min to 100°C, then programmed from 100°C to 200°C at 25°C/min. The injector was maintained at 250°C. Alcohol compounds were isolated by ethyl acetate extraction. A 1 μl sample was injected in split injection mode with a 20:1 split ratio.

### Analysis of isoprenol and prenol by GC–FID

The produced isoprenol and prenol were quantified by a GC equipped with flame ionization detector (FID). The separation of isoprenol and prenol was performed using a CP-FFAP CB capillary column (25 m × 0.25 mm; 0.2 μm film thickness) purchased from Agilent Technologies (Santa Clara, CA). The oven temperature was initially held at 50°C for 1 min, then raised with a gradient of 5°C/min until reaching 100°C, and finally programmed to 150°C at 25°C/min. Nitrogen was used as the carrier gas. The injector and detector were held at 250°C and 270°C, respectively. Samples were prepared by ethyl acetate extraction. Isoamyl alcohol was added into the samples as the internal standard before solvent extraction.

## Abbreviations

IPP: Isopentenyl pyrophosphate; DMAPP: Dimethylallyl pyrophosphate; IPTG: Isopropyl β-D-thiogalactoside; PCR: Polymerase chain reaction; GC: Gas chromatography; FID: Flame ionization detector; GC-MS: Gas chromatography–mass spectrometry.

## Competing interests

The authors declare that they have no competing interests.

## Authors’ contributions

YZ designed the research and prepared the manuscript. MX and WQ helped to revise the manuscript. YZ, QL and LL did the lab work, plasmid construction, site-directed mutagenesis, strain cultivation and product detection. JY, HZ, XJ and TC did some work in plasmid construction. WL and XX did some work in product detection. All authors read and approved the final manuscript.
